# Smooth to Rough Dissociation in *Brucella*: The Missing Link to Virulence

**DOI:** 10.3389/fcimb.2015.00098

**Published:** 2016-01-05

**Authors:** Marcos Mancilla

**Affiliations:** Research and Development Department, ADL Diagnostic Chile Ltd.Puerto Montt, Chile

**Keywords:** *Brucella*, lipopolysaccharide, O-polysaccharide, rough, smooth

## Abstract

Dissociation encompasses changes in a series of phenotypes: colony and cell morphology, inmunological and biochemical reactions and virulence. The concept is generally associated to the *in vitro* transition between smooth (S) and rough (R) colonies, a phenotypic observation in Gram-negative bacteria commonly made since the beginning of microbiology as a science. It is also well known that the loss of the O-polysaccharide, the most external lipopolysaccharide (LPS) moiety, triggers the change in the colony phenotype. Although dissociation is related to one of the most basic features used to distinguish between species, i.e., colony morphology, and, in the case of pathogens, predict their virulence behavior, it has been considered a laboratory artifact and thus did not gain further attention. However, recent insights into genetics and pathogenesis of members of *Brucella*, causative agents of brucellosis, have brought a new outlook on this experimental fact, suggesting that it plays a role beyond the laboratory observations. In this perspective article, the current knowledge on *Brucella* LPS genetics and its connection with dissociation in the frame of evolution is discussed. Latest reports support the notion that, by means of a better understanding of genetic pathways linked to R phenotype and the biological impact of this intriguing “old” phenomenon, unexpected applications can be achieved.

The *Brucella* genus includes Gram-negative microorganisms that cause brucellosis, a major worldwide zoonosis. The taxonomical criteria used to divide the genus into several species include host preference, physiological differences, phage susceptibility and cell envelope structural features. Based on the aspect of colonies on agar plates, which is in accordance with the cell surface and lipopolysaccharide (LPS) structure, *Brucella* may occur either as smooth (S) or rough (R) species. The zoonotically more relevant S species *B. melitensis, B. suis* and *B. abortus* express a full LPS molecule (S-LPS) that is anchored in the outer membrane (OM) (Whatmore, 2009). This group furthermore comprises species that have been isolated from rodents (*B. neotomae*) and marine mammals (*B. ceti* and *B. pinnipedialis*; Foster et al., 2007). More recently, *B. microti*, primarily isolated from voles and red foxes, was isolated directly from soil, a fact that has not been reported for any other S species (Scholz et al., 2008). In contrast, the naturally occurring R species *B. ovis* and *B. canis* express R-LPS that lacks the O-antigen, a trait linked to their reduced virulence.

## Brucella LPS: Overall structure and function

**Figure 1:**
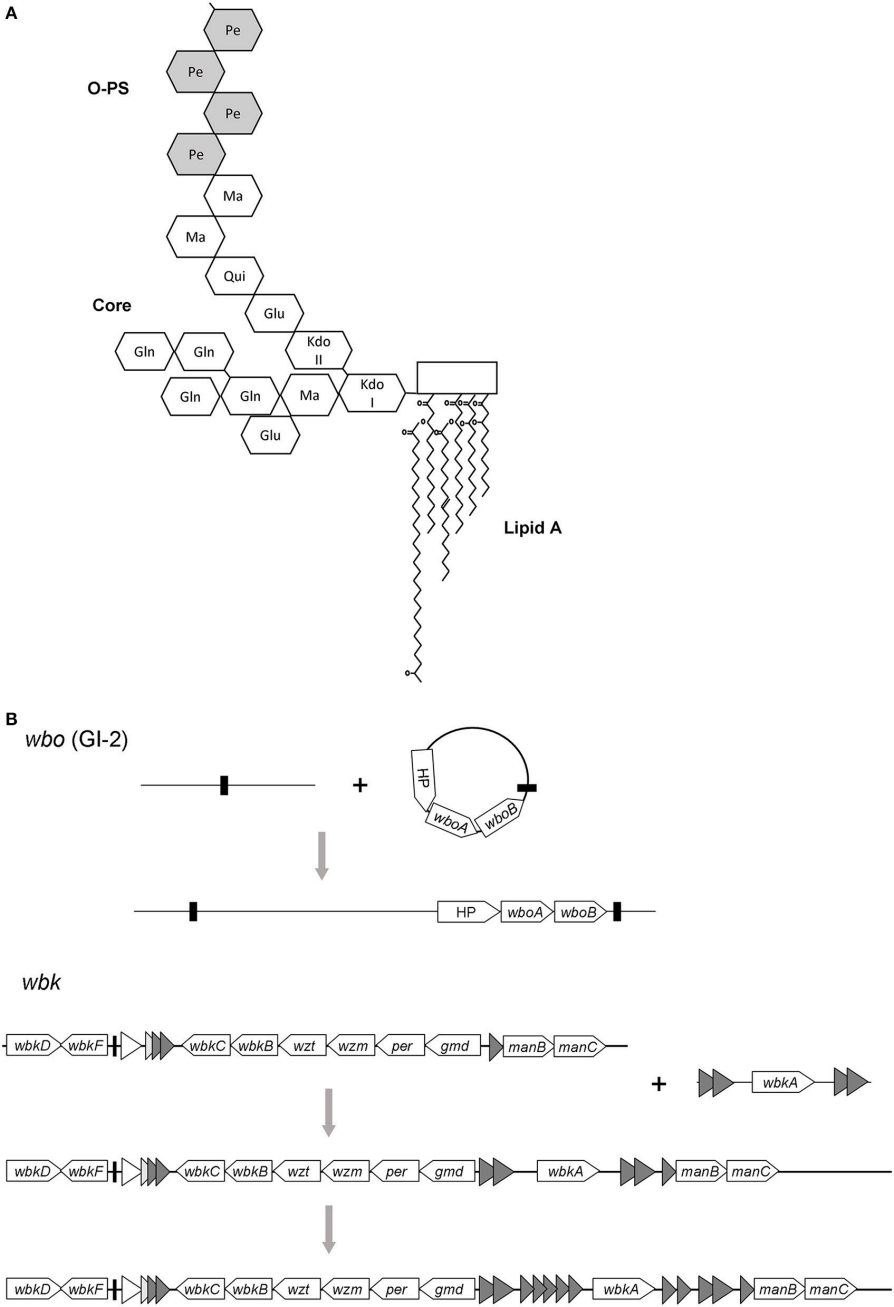
**(A)** A summary of *Brucella* LPS structure showing the sugar backbone of O-PS and core plus lipid A. Glu, glucose; Gln, glucosamine; Ma, mannose; Pe, N-formylperosamine; Qui, quinovosamine (based on Iriarte et al. (2004) and Gil-Ramírez et al. (2014). **(B)** Evolutionary scenario proposed for the O-polysaccharide in *Brucella*. The *wbo* locus was probably acquired by a single horizontal transfer event involving the unstable element GI-2 and its cognate integrase. In contrast, the *wbk* locus might evolve from a primary integration, followed by the addition of the *wbkA* gene by transposition. Thus, the *wbkA* region could serve as bait for transposition of related elements (shaded triangles). Black bars indicate direct repeats and a tRNA gene in *wbo* and *wbk* regions, respectively.

Similar to many LPS of Gram-negative pathogens, the *Brucella* LPS plays a fundamental role in the interaction with the corresponding host. In contrast to the well-known endotoxic properties manifested by enterobacterial LPS, the *Brucella* LPS is a paradigm of a poorly endotoxic, barely proinflammatory molecule unable to activate the innate immunity to a great extent (Moreno et al., 1981; Lapaque et al., 2005). Its structure consists of three covalently bound canonical motifs anchored in the OM: lipid A, the most hydrophobic part that is embedded in the OM; a core oligosaccharide that creates a bridge to the O-antigen or O-polysaccharide (O-PS), the third and most external surface moiety. *Brucella* lipid A contains a diaminoglucose backbone with reduced quantities of phosphate. The backbone is substituted with saturated C16 and C18 fatty acids, but also with unusual long-chain hydroxilated C28 and other very long acyl chains, a feature that *Brucella* share with members of the plant-symbiont genus *Rhizobium* (Moreno et al., 1990; Iriarte et al., 2004). The lipid A is linked to an oligosaccharide core chemically composed of 3-deoxy-D-manno-2-octulosonic acid (KDO), glucosamine, glucose, mannose and quinovosamine (Iriarte et al., 2004). Even though the core structure has not yet been definitively solved, recent insights suggest that it holds a mannose-containing lateral branch that hampers the recognition by complement, antimicrobial peptides and pathogen recognition receptor complex TLR4-MD2 (Conde-Álvarez et al., 2012). In addition, genetic and structural analyses demonstrated that some core sugars are not connecting the O-PS with the lipid A, which confirms such a branched array (Conde-Álvarez et al., 2012; Kubler-Kielb and Vinogradov, 2013; Gil-Ramírez et al., 2014). The O-PS is a linear homopolymer of N-formylperosamine, an unusual chemical composition among members of α-2 Proteobacteria (Moreno and Moriyón, 2006). The O-PS confers resistance to the innate bactericidal response by preventing deposition of complement factors at the cell surface (Eisenschenk et al., 1999), but also by impairing the binding of antimicrobial peptides to the membrane (Martínez de Tejada et al., 1995). The O-PS also hinders the production of proinflammatory cytokines (Barquero-Calvo et al., 2007), and along with the core provides receptor moieties for brucellaphages (Monreal et al., 2003). Hence, the interference with innate immunity mechanisms induced by *Brucella* LPS is critical to avoid an early host immune response, thus allowing a successful intracellular infection (Gorvel and Moreno, 2002). A scheme of LPS structure is given in Figure 1A.

## Horizontal transfer and O-PS acquisition

Contrary to the structure of lipid A and core of LPS, the O-PS is quite variable in bacteria. The diversity of repeat units within the O-PS and linkages between them is responsible for the O-PS variation and provides the basis for the O-serotyping classification in *Enterobacteriaceae* (Wang et al., 2010). The genes responsible for the O-PS synthesis in Gram-negative bacteria are mainly clustered in the chromosome and often form a single transcriptional unit (Reeves and Wang, 2002). They can be categorized into three groups: nucleotide sugar pathway genes; those encoding glycosyltransferases (GT), which can also be found scattered throughout the genome; and those for processing and transport. *Brucella* is no exception from this rule, its O-PS genes are encoded in two main loci *wbk* and *wbo*. The O-PS synthesis depends on two GT genes carried by *wbo* (*wboA* and *wboB*) (McQuiston et al., 1999; González et al., 2008) that are included in the *Brucella* genomic island GI-2 (Rajashekara et al., 2008), an unstable genetic element of 15.1 kb (Mancilla et al., 2010). This region carries an additional gene, located close to GT genes, that encodes a hypothetical protein (BMEI0999) that apparently is part of the O-PS synthetic machinery since attempts to complement ΔGI-2 mutants using a plasmid carrying only *wboA-wboB* have failed (Rajashekara et al., 2008). Major O-PS locus *wbk* is located between a ribose transport system (*rbs*) and an *rnc* gene (Moriyón et al., 2004). The low GC content of approximately 50%, a feature shared with many O-PS clusters, has been linked to its hypothetical acquisition via horizontal transfer (Cloeckaert et al., 2000; Godfroid et al., 2000). This region encodes the genes putatively necessary to synthesize perosamine (*gmd, per*), n-formylation of perosamine residues (*wbkC*), GT for polymerization (*wbkE, wbkA*), to prime bactoprenol (*wbkD, wbkF*), and ABC transporters that translocate the O-PS (*wzm* and *wzt*). Genes for mannose synthesis have also been identified in the same region (*manA*_OAg_, *manB*_OAg,_ and *manC*_OAg_), but mutational analysis of *manB*_OAg_ indicated that it is not essential (González et al., 2008), since independent homologs located in the chromosome II (*manBA*_core_) are able to meet mannose demands (Monreal et al., 2003). To date, there is no confirmed function for *wbkB* (encodes a putative perosamine synthetase) because the corresponding mutant preserved the S phenotype and the hypothetical ligase that binds the amino sugar O-PS to the lipid A-core in the periplasmic interface has not yet been identified (Moriyón et al., 2004).

Concerning horizontal acquisitions outside from *wbk* and *wbo*, the role of a gene cluster encoding enzymes for LPS biosynthesis has been proposed (Vizcaíno et al., 2001). The cluster was named GI-8 and can be found in the majority of “classic” *Brucella* species although it is absent from *B. abortus* (Rajashekara et al., 2004). Consistent with their functionality, the expression of several genes carried by GI-8 has been detected in *B. melitensis* (Rossetti et al., 2009). Moreover, an exopolysaccharide consisting of glucosamine, glucose and mostly mannose has been described in *B. melitensis* 16M (Godefroid et al., 2010). The annotation matches with genes expected for the biosynthesis of constituents previously mentioned, therefore the data strongly support a role of GI-8 in the production of such an exopolysaccharide.

Since members of *Brucella* are facultative intracellular parasites that usually inhabit a constrained environment that precludes horizontal transfer with other bacteria, this ecological niche might explain the homogeneity of their O-PS structures or, in other terms, the restricted O-serotyping diversity found across *Brucella* species. This point of view does not contradict the acquisition of O-PS genes via HGT because it is thought that they were captured and integrated into the ancestral *Brucella* genome before the speciation (Figure 1B).

## Mechanisms of dissociation

The phenotypic change from S to R colonies experienced by *Brucella* under cultivation is widely known (Braun, 1946). Nevertheless, the S-R dissociation is far from being a behavior exclusively manifested by the brucellae. Several reports (McCallum et al., 1989; Liu and Reeves, 1994; Walsh and Moran, 1997) and reviews (Reeves, 1995; Reeves and Wang, 2002; Wang et al., 2010) have reported on this phenomenon in major *Enterobacteriaceae* species and even in microorganisms phylogenetically remote such as *Mycobacteriaceae* (Eckstein et al., 2000). It has been speculated that the propensity to become rough under laboratory conditions reflects the fact that the O-PS is needed in natural niches (Reeves, 1995). Indeed, mutants defective in O-PS are generally serum sensitive, since their lipid A represents an exposed target for complement killing activity (Rautemaa and Meri, 1999). Despite its importance in virulence for Gram-negative pathogens, the mechanisms affecting the stability of O-PS, i.e., dissociation, have only been marginally explored. In *Brucella*, this phenomenon has been described very early (Henry, 1933; Braun, 1946) and is of great importance in basic research and vaccine production (Alton et al., 1988). Furthermore, the organization of its O-PS clusters and the location of the O-PS GT is representative of several Gram-negative bacteria and, therefore, clues from *Brucella* dissociation mechanisms may be extrapolated to other bacteria.

According to the stochasticity of the mutations implicated in dissociation, we can distinguish two main pathways. There are unpredictable mutations in which the LPS gene(s) affected originate from an apparently coincidental event. In contrast, mutations that arise upon a discrete and reproducible recombination event follow well-defined pathways. Typical examples for random mutations are those displayed by attenuated vaccine strains obtained through several culture passages (Alton et al., 1988; Moriyón et al., 2004). In some instances, the corresponding R-linked genetic defect could be identified as was the case with the *B. abortus* RB51 strain. Transposition of the IS*711* element into the *wboA* caused the disruption of the gene, a fact that contributed to the roughness of the RB51 strain (Vemulapalli et al., 1999). In *B. melitensis* B115 R strain, a nonsense mutation in *wzm* was identified that interrupts the O-PS transport and thus causes accumulation of O-PS in the cytoplasm of this mutant (Adone et al., 2008). Further reports have shown that point mutations, indels and rearrangements of the *manBA*_core_ locus occurred in spontaneous *B. melitensis* 16M R mutants (Turse et al., 2011) and that R strains maintained in the laboratory over years can accumulate mutations on LPS genes (Adone et al., 2011).

It has become clear that *Brucella* and other organisms carry unstable, mobile elements implicated in chromosomal deletions that lead to the loss of LPS genes. These DNA fragments are often maintained under selective pressure from specific exposure to the host, which means that upon environmental changes they can be released from the chromosome. This statement especially applies for brucellae, whose O-PS loci are located in spots of recombination. For instance, the *wbo* locus can spontaneously excise from the chromosome due to being part of a genomic island (Mancilla et al., 2010). The excision is prompted by site-specific recombination between 41 bp flanking repeats catalyzed by the phage-related integrase of GI-2 (Mancilla et al., 2010). Interestingly, this mechanism, along with spontaneous mutations in *wbk* genes (Zygmunt et al., 2009), could be involved in the evolution of the naturally R species *B. ovis* that lacks GI-2 (Vizcaíno et al., 2004). The role of homologous recombination mediated by the RecA protein in dissociation has also been investigated (Mancilla et al., 2012). This recombination activity is responsible for the spontaneous excision of *wbkA*, which resides in a putative transposon remnant. *wbkA*- flanking IS*Bm1* elements are involved in a recombination event that leads to the excision and loss of a 5.5 kb fragment including the *wbkA* gene from a small fraction of the bacterial population. It must be pointed out that the excessive recombination affecting these loci has been observed under unfavorable culture conditions, but the biological significance remains unclear. On the other hand, a deletion of 351 bp comprising the *wbkF-wbkD* genes has given rise to the R phenotype of *B. canis* (Zygmunt et al., 2009). The mutation might have occurred due to a slipped mispairing mechanism involving short direct repeats. Although, it may follow a reproducible pathway, the deletion has not been detected in samples positive for GI-2 and *wbkA* deletions (unpublished results). The genetic events related to *Brucella* dissociation are summarized in Table 1.

**Table 1:** **Stochastic and non-stochastic events related to dissociation**.

**S-R mutation**	**Mechanism**	**Species/strain**	**References**
GI-2 deletion	Site-specific recombination	*B. abortus, B. melitensis*, and *B. suis*	Mancilla et al., 2010
*wbkA* deletion	Homologous recombination	*B. abortus, B. melitensis*, and *B. suis*	Mancilla et al., 2012
*wbkFD* deletion	Strand-slippage during replication	*B. canis*	Zygmunt et al., 2009
*manBA*_core_ indels, large deletion	Strand-slippage, homologous recombination	*B. abortus* 2308, *B. melitensis* 16M	Turse et al., 2011
*wboA*::IS*711*	Gene disruption by IS transposition	*B. abortus* RB51	Vemulapalli et al., 1999
*wzm* mutation	Frameshift derived from a point mutation	*B. melitensis* B115	Adone et al., 2011

In this context, the repeat units found in the *wbk* region deserve special attention. The presence of IS and related remnants may offer sequence substrates for homologous recombination which could result in extensive deletions. Indeed, the atypical *Brucella* spp. BO2 strain seems to have lost a large portion of the *wbk* locus. Instead of that, a cluster of rhamnose-based O-PS biosynthetic genes is responsible for the S-LPS phenotype depicted by this strain, which explains the untypeable character using conventional antibodies (Wattam et al., 2012; Zygmunt et al., 2012).

## The biological significance of dissociation

It has been stated that *Brucella* O-PS is a critical virulence factor of classical S species. Spontaneous R variants undergo enhanced intracellular killing by macrophages (Fernandez-Prada et al., 2001), consistent with an increased activation of these cells in culture (Fernandez-Prada et al., 2003; Rittig et al., 2003). In the murine brucellosis model, R mutants are cleared faster than their S counterpart (Allen et al., 1998). We know that the O-PS is largely responsible for the stealthy behavior manifested by the S cells, which is why it does not seem likely that dissociation plays a role under field conditions, where the pressure imposed by the host limits growth and spread. However, in a recent study concerning the LPS expression of *B. melitensis* in infected cell cultures, it has been shown that it is possible to isolate R types from mice infected with S cells (Turse et al., 2011). The authors argued that dissociation is a natural process taking place during infection, a conclusion also supported by the enhanced growth shown by the *manBA*_core_ mutants recovered. Later, the same research team demonstrated that the cytotoxicity of R cells are necessary for *in vitro* egress and dissemination of *B. melitensis* S cells from infected host cells, suggesting for the first time a biological role for dissociation (Pei et al., 2014). Contrary to what would have been expected, this fact might change the current model of infection in which the S cells are able to induce cell lysis and spread by themselves, without relying on R mutants (Starr et al., 2012). As a consequence, we can speculate that this finding would also explain the presence of R strains originated from S species in collections of field strains (Dorneles et al., 2014; Bertu et al., 2015).

Examples of genomic changes that impact the infection outcome suggest that there are loci, mainly encoding virulence genes, that are preferentially mutated and even lost during the infection. These mutations may lead to lifelong host-pathogen relationship, a fact that has already been described for some uropathogenic *Escherichia coli* strains (Zdziarski et al., 2010). Interestingly, spontaneous mutations on LPS genes of *Burkholderia pseudomallei*, causative agent of melioidosis, have been proposed to be involved in the persistence of some R strains (Tuanyok et al., 2012). Moreover, *B. melitensis* R variants have been ocassionally isolated from goat milk samples, suggesting that R types can survive in the mammary gland (Mancilla et al., 2012). It might be inferred that only those mutations that originate R types with a balanced intracellular fitness that allows competition with S cells would prevail. Therefore, the abolition of dissociation mechanisms or pathways predicts a deleterious effect on the fitness of the resulting R-mutants that may impact the infection outcome. If S brucellae have evolved into dissociation-prone species able to change their major “pathogen credential” when necessary is yet a matter of speculation. But certainly, the emergence of R types *in vitro* as well as *in vivo* suggests that brucellae may not only experience phenotypic heterogeneity but also that this fact could be needed for displaying full virulence of S cells. The idea that dissociation may occur in the extracellular milieu is stressed by the recent finding of *B. microti* environmental isolates devoid of O-PS (Al Dahouk et al., 2012). Aditionally, reversion (from R to S) has been reported *in vivo*, a fact that accounts for the mentioned heterogeneity during host-pathogen interaction (Pérez-Sancho et al., 2014). But this phenomenon, that suggests reversibility of dissociation, still awaits for further genetic characterization.

## Future directions

The acquisition of clusters of genes is recognized as the major driving force for prokaryotic evolution (Hacker and Carniel, 2001). These clusters are part of the flexible, not essential gene pool that is maintained due to the environmental pressure, the integrative machinery encoded by the foreign fragment and/or endogenous homologous recombination (Gal-Mor and Finlay, 2006). Once the selective pressure is removed, spontaneous gene or even cluster deletions are triggered by recombination often by the system involved in the chromosomal integration process. The result of such deletions is a dramatic loss of virulence of the pathogen and over-attenuation in the case of vaccine strains. An important example concerning the impact of excessive recombination on the stability of vaccine strains is *M. bovis* BCG, the strain used to prevent tuberculosis. In fact, the deletion of a single 9 kb fragment known as RD1 is responsible for the marked attenuation of BCG in comparison with *M. tuberculosis* and *M. bovis* pathogenic strains (Pym et al., 2002).

In the case of *Brucella*, the licensed *B. abortus* S19 and *B. melitensis* Rev 1 s strains are superior vaccines with a long history of successful utilization in the eradication of brucellosis but unfortunately tend to dissociate, a characteristic related to a low efficacy (Alton et al., 1988; Grilló et al., 2000). The discovery of genes and *cis* elements involved in LPS-loci deletion pathways prepare the ground to control undesirable excessive recombination events and thus to avoid over-attenuation of established S live vaccines. A proof of this concept is the recent development of an improved version of *B. melitensis* Rev 1. Interestingly, the newly engineered strain called Rev 2 exhibits an enhanced stability owing to the abolishment of two well-proven dissociation mechanisms (Mancilla et al., 2013). Noteworthy, similarities between *Brucella* LPS loci organization and other Gram-negative bacteria suggest that those may also have acquired their O-PS by horizontal transfer and might undergo the same dissociation mechanisms. Therefore, awareness of these processes could be generally useful to obtain more stable bacterial strains for antigen and vaccine production, regarding not only control and eradication of brucellosis: this knowledge may be applied to improve vaccines against a broader spectrum of bacterial infections. However, dissociation pathway(s) to be abrogated should be carefully studied in order to avoid disruption of mechanisms important in the context of infection and development of a specific immunity.

### Conflict of interest statement

The author declares that the research was conducted in the absence of any commercial or financial relationships that could be construed as a potential conflict of interest.
